# Endoplasmic Reticulum Remodeling Tunes IP_3_-Dependent Ca^2+^ Release Sensitivity

**DOI:** 10.1371/journal.pone.0027928

**Published:** 2011-11-30

**Authors:** Lu Sun, Fang Yu, Aman Ullah, Satanay Hubrack, Arwa Daalis, Peter Jung, Khaled Machaca

**Affiliations:** 1 Department of Physiology and Biophysics, Weill Cornell Medical College in Qatar, Education City – Qatar Foundation, Doha, Qatar; 2 Department of Physics and Astronomy and Quantitative Biology Institute, Ohio University, Athens, Ohio, United States of America; University of Queensland, Australia

## Abstract

The activation of vertebrate development at fertilization relies on IP_3_-dependent Ca^2+^ release, a pathway that is sensitized during oocyte maturation. This sensitization has been shown to correlate with the remodeling of the endoplasmic reticulum into large ER patches, however the mechanisms involved are not clear. Here we show that IP_3_ receptors within ER patches have a higher sensitivity to IP_3_ than those in the neighboring reticular ER. The lateral diffusion rate of IP_3_ receptors in both ER domains is similar, and ER patches dynamically fuse with reticular ER, arguing that IP_3_ receptors exchange freely between the two ER compartments. These results suggest that increasing the density of IP_3_ receptors through ER remodeling is sufficient to sensitize IP_3_-dependent Ca^2+^ release. Mathematical modeling supports this concept of ‘geometric sensitization’ of IP_3_ receptors as a population, and argues that it depends on enhanced Ca^2+^-dependent cooperativity at sub-threshold IP_3_ concentrations. This represents a novel mechanism of tuning the sensitivity of IP_3_ receptors through ER remodeling during meiosis.

## Introduction

The egg-to-embryo transition marks the initiation of multi-cellular organismal development and is instigated by a series of cellular events following fertilization collectively referred to as egg activation [1]. These events are encoded in a sequential fashion by the fertilization-induced Ca^2+^ transient, which possesses specialized spatial and temporal dynamics that are necessary and sufficient for egg activation [2]. The egg acquires the competency to produce the fertilization-specific Ca^2+^ signal during oocyte maturation, a complex developmental pathway that prepares the egg for fertilization. Oocyte maturation is driven by multiple kinase cascades induced downstream of the hormonal signal that releases meiotic arrest [3]. The dynamic remodeling of Ca^2+^ signaling is fundamental to the developmental competence of the egg [2]. It further elegantly illustrates the versatility of Ca^2+^ signals given the broad bandwidth inherent in their temporal and spatial features. This results in an information rich signal that instructs multiple cellular events at egg activation, including the block to polyspermy and completion of meiosis [2].

At steady state IP_3_ receptors in different cell types cluster in a hierarchical fashion leading to Ca^2+^ release events of varying sizes, referred to as blips for the smallest events, presumably due to the opening of a single IP_3_ receptor, or puffs for larger events [4]–[6]. Such elementary events coalesce to produce global Ca^2+^ release waves [7]–[9]. In addition, studies have shown that sustained IP_3_ or Ca^2+^ levels lead to a slow aggregation of IP_3_ receptors into large clusters independently of ER structure, presumably through protein-protein interactions [10]–[12]. Functionally IP_3_ receptor clustering allows for a continuum of the IP_3_-dependent Ca^2+^ release events that serve specific cellular needs.

An important aspect of Ca^2+^ signaling differentiation during oocyte meiosis that is conserved among different species, is the increased sensitivity of IP_3_-dependent Ca^2+^ release [13]–[15]. Ca^2+^ release through IP_3_ receptors is a major contributor to the fertilization-induced Ca^2+^ signal in vertebrates [16]. The enhanced sensitivity of IP_3_-dependent Ca^2+^ release is often described as a larger Ca^2+^ transient in eggs following store mobilization [14], [17], [18]. However this does not necessarily translate into sensitization of IP_3_ receptors per se, since additional factors could contribute to the duration and amplitude of the Ca^2+^ release signal, including Ca^2+^ extrusion and influx, cytosolic buffering capacity, and Ca^2+^ reuptake into stores. A more accurate description of IP_3_ receptor sensitization is reflected as Ca^2+^ release in the egg at lower threshold IP_3_ concentrations that induce minimal or no release in the immature oocyte [13], [15]. Also noteworthy is the fact that additional Ca^2+^ signaling pathways are modulated during meiosis, including inhibition of Ca^2+^ influx, internalization of the plasma membrane Ca^2+^-ATPase, and Ca^2+^ recycling between the cytoplasm and ER lumen due to prolonged opening of IP_3_ receptors [19]–[23]. Collectively modulation of these different Ca^2+^ signaling modules defines the dynamics of the fertilization-induced Ca^2+^ transient.

Enhanced Ca^2+^ release in the egg correlates with IP_3_ receptor phosphorylation, and is clearly dependent on the activation of the oocyte maturation kinase cascades [15], [24], [25]. Although this correlation postulates a direct role of IP_3_ receptor phosphorylation in the sensitization of Ca^2+^ release [24]–[26], this remains to be shown. A potential mechanism that could contribute to IP_3_ receptor sensitization is increased affinity of the IP_3_ receptor [27]. However, this has not been proven experimentally, highlighting the fact that the mechanistic regulation of IP_3_-dependent Ca^2+^ release sensitization during meiosis remains largely undefined. In addition, the number of functional IP_3_ receptors increases during *Xenopus* oocyte maturation following the disruption of annulate lamellae, which are specialized membranous structures where the activity of a sub-population of IP_3_ receptors is inhibited [28]. This inhibition is due to nuclear pore complexes, which are enriched in annulate lamellae as a molecular stockpile for embryonic divisions [29].

Furthermore, alterations to IP_3_-dependent Ca^2+^ release have been shown to correlate with ER remodeling in oocytes from several species [30]. In *Xenopus* ER patches nucleate Ca^2+^ waves and patch appearance coincides with IP_3_-dependent Ca^2+^ release sensitization [17], [28]. Similarly in mouse eggs the Ca^2+^ wave pacemaker localizes spatially with ER patches, and ER remodeling correlates with modulation of IP_3_-dependent Ca^2+^ release [31], [32]. This has led to the suggestion that the physical clustering of the ER contributes to the greater sensitivity to IP_3_ in the mature egg [30], however the mechanisms underlying such a role for ER remodeling are not known. Data presented herein provide a mechanistic explanation for the correlation between ER remodeling and IP_3_-dependent Ca^2+^ release sensitization. IP_3_ receptors enriched in ER patches respond to sub-threshold IP_3_ concentrations that do not produce Ca^2+^ release in the neighboring reticular ER, showing that IP_3_-dependent Ca^2+^ release is sensitized in ER patches. We present evidence that this sensitization in ER patches is due to enhanced Ca^2+^-dependent cooperativity between receptors due to the increased density of IP_3_ receptors within an ER patch.

## Results

### ER remodels during meiosis

**Figure 1:**
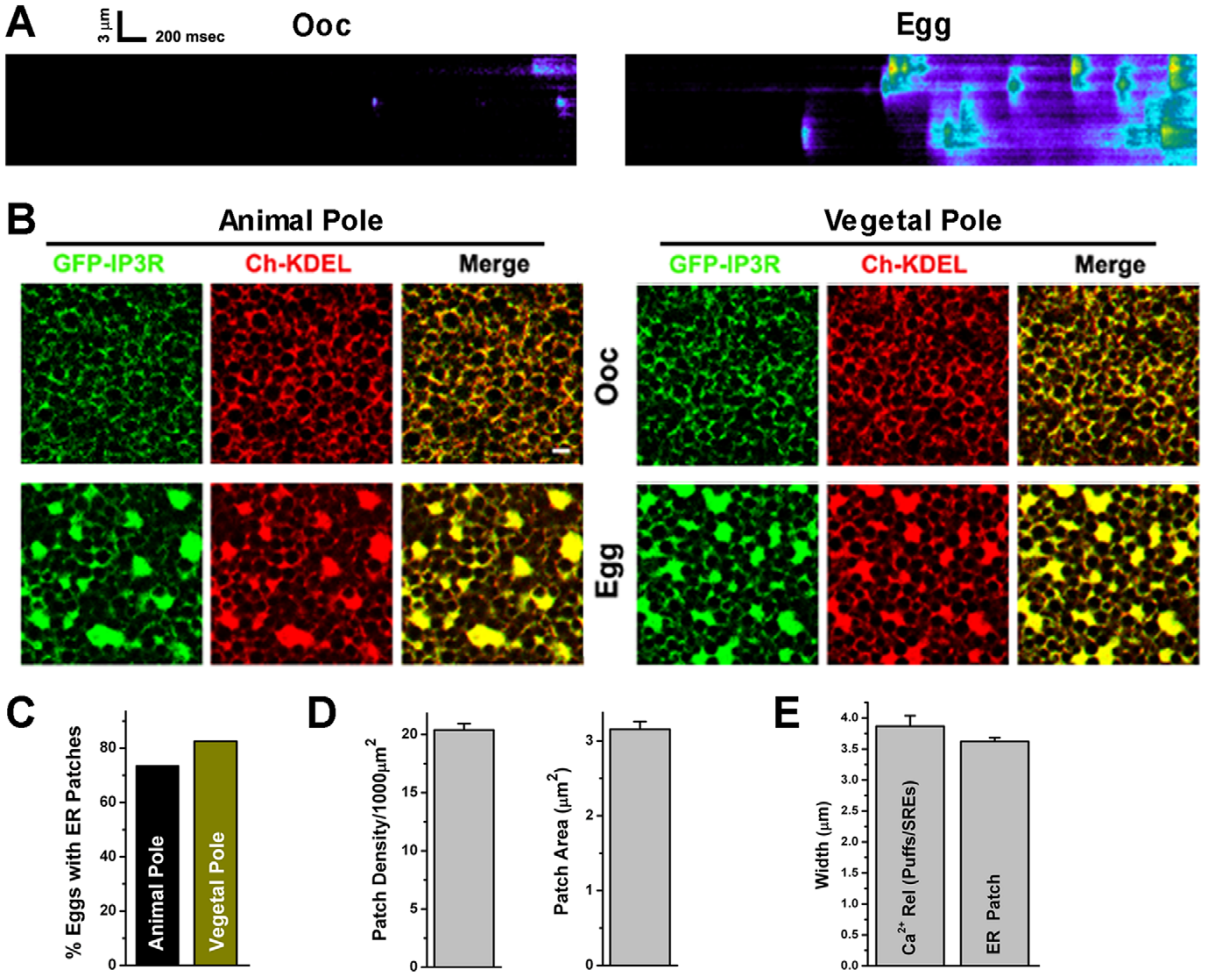
ER remodeling and IP_3_ receptor clustering during meiosis. **A.** **Functional clustering of elementary Ca^2+^ release events during oocyte maturation.** *Xenopus* oocytes were injected with 10 µM caged IP_3_ and 40 µM Oregon-green. Oocyte maturation was induced with progesterone and both immature oocytes and fully mature eggs were imaged in linescan mode at 488 nm with the 405 nm laser at low intensity (0.2%) to continuously uncage cIP_3_. The same region in the cell was scanned continuously in linescan mode with the x-axis representing time and the y-axis space. The single isolated Ca^2+^ puffs observed in the oocyte coalesce into larger release events referred to as single release events (SRE). **B.** **The ER remodels during oocyte maturation to form large patches in the egg.** Oocytes were injected with GFP-IP_3_ receptor (IP_3_R) (50 ng/cell) and mCherry-KDEL (10 ng/cell). Images show the formation of ER patches in both animal and vegetal hemisphere to which IP_3_ receptors localize (Scale bar, 2 µm). **C.** Frequency of ER patches on the animal and vegetable poles (n = 166; 14 frogs). **D.** ER patch density and area (n = 38). **E.** Width of elementary Ca^2+^ release events in the egg as compared to the width of ER patches.

**Figure 2:**
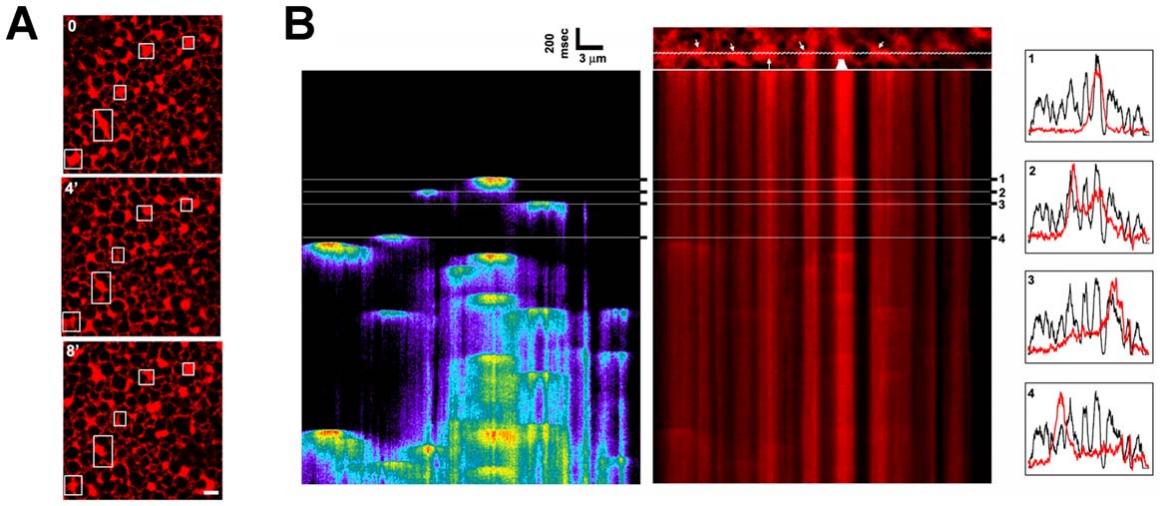
A. ER patches remodel continuously. Examples of images from a time series from an egg expressing mCherry-KDEL (Scale bar, 2 µm). **B.** **Elementary Ca^2+^ release events in the egg localize to ER patches.** mCherry-KDEL expressing eggs injected with cIP3 and Oregon-green were line-scanned with the 405 nm laser on (0.2%). Left image show Ca^2+^ release events and the right ER distribution. The xy image with the linescan area is shown on top of the ER linescan. Histograms of Ca^2+^ release (red trace) and ER distribution (black trace) are shown for four selected areas as indicated by the numbers.

We have previously shown that elementary IP_3_-dependent Ca^2+^ release events (Ca^2+^ puffs) cluster dramatically during oocyte maturation, and that this clustering is important for the mode and speed of Ca^2+^ wave propagation in the egg [15]. Whereas Ca^2+^ puffs in the oocyte are separate, they coalesce in the egg following maturation (Fig. 1A). Ca^2+^ transients were imaged in line scan mode on the animal hemisphere while continuously uncaging IP_3_ using the near UV 405 nm laser at low intensity (Fig. 1A). This allows for gradual build-up of IP_3_ concentration thus inducing Ca^2+^ puffs (Fig. 1A). Note the spatially overlapping Ca^2+^ puffs in the egg (Fig. 1A). Because ER remodeling was reported to be limited to the vegetal hemisphere of the egg [17], we initially interpreted the clustering of Ca^2+^ puffs on the animal hemisphere as being due to lateral diffusion of IP_3_ receptors in the plane of the ER membrane to form overlapping Ca^2+^ release sites [15]. Attempts to visualize such IP_3_ receptor clusters in eggs expressing GFP-tagged IP_3_ receptor were unsuccessful. Rather we observed large three dimensional aggregations of IP_3_ receptors that were reminiscent of ER patches observed during maturation (Fig. 1B) [17]. Indeed these IP_3_ receptor aggregations co-localize with the ER marker, mCherry-KDEL (Fig. 1B). In the immature oocyte both the IP_3_ receptor and ER have a reticular distribution, that remodels into a combination of reticular and large ER ‘patches’ during oocyte maturation (Fig. 1B, Egg). Contrary to what has been previously reported [17], ER reorganization is observed on both the animal and vegetal poles of the egg (Fig. 1B). We assessed ER remodeling following oocyte maturation in 166 eggs from 14 donor females using various markers for the ER, including the IP_3_ receptor, STIM1, GFP-KDEL and mCherry-KDEL. In all cases we observed ER remodeling on the animal hemisphere. Although ER patches are observed at a slightly lower frequency on the animal pole, with pronounced cell to cell variability, they are present in most eggs (73.5%) (Fig. 1C). ER patch density was ∼20/1000 µm^2^ with an average area of 3.1+0.087 µm^2^ (Fig. 1D), consistent with a previous report [17], but not others [28], [29]. Nonetheless, this only provides a snapshot of ER remodeling, since ER patches in the egg are dynamic motile structures that are continuously restructured (Figure 2A). Furthermore, the steady state distribution of ER patches shows a more cortical residence in the vegetal compared to the animal hemisphere (Fig. S1A), and their formation depends on the microtubule network, since 75% of oocytes (n = 36) treated with nocodazole did not form ER patches during maturation (Fig. S1B).

### ER remodeling into large patches sensitizes IP_3_-dependent Ca^2+^ release

The fact that ER remodeling was observed on both the animal and vegetal hemispheres argues that Ca^2+^ puffs clustering during meiosis is not due to lateral diffusion of IP_3_ receptors, but rather to ER remodeling. This is supported by the similar width of ER patches and that of elementary Ca^2+^ release events (SREs) in eggs (Fig. 1E). In addition, if ER remodeling underlies Ca^2+^ puff redistribution, then Ca^2+^ release activity should coincide spatially with ER patches. This is indeed the case as shown in Figure 2B. Gradual uncaging of IP_3_ results, after a time lag, in Ca^2+^ release SREs that coalesce into a Ca^2+^ wave (Fig. 2B). Cross sectional profiles of both ER distribution (black) and Ca^2+^ release activity (red) demonstrate co-localization of individual SREs to ER patches (Fig. 2B). This co-localization was observed consistently in at least 15 cells tested and shows that elementary Ca^2+^ release events in the egg localize to ER patches.

IP_3_ receptors localize to both the reticular ER and ER patches in the egg (Fig. 1B), yet initial Ca^2+^ release events are observed preferentially within ER patches (Fig. 2B) [28]. This argues for a differential sensitivity of IP_3_ receptors based on the ER sub-domain they localize to. To test whether this is the case we undertook of series of functional experiments to directly assess Ca^2+^ release sensitivity in the different ER spatial domains.

**Figure 3:**
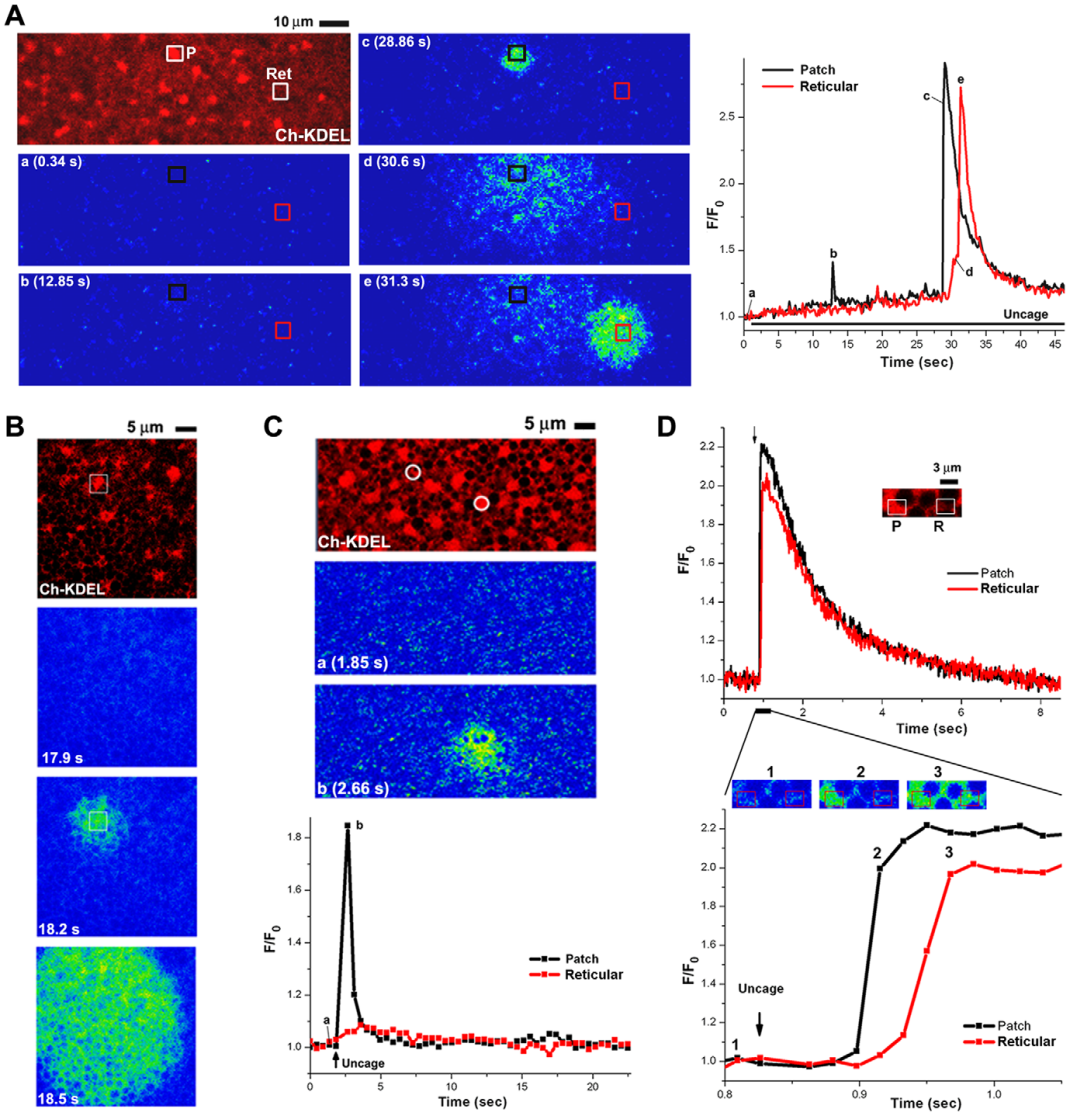
Sensitization of Ca^2+^ release in ER patches. Eggs expressing mCherry-KDEL were injected with cIP_3_, and Oregon-green and imaged ∼1 hr after GVBD. **A–B.** **IP_3_ receptors in ER patches respond to IP_3_ concentrations not detected by receptors in the reticular ER.** ER distribution and Ca^2+^ release were imaged over time, while continuously uncaging cIP_3_ only within two ROIs as indicated by the boxes using the 405 nm laser. One ROI was placed over an ER patch (P) and the other over a reticular ER domain (Ret). Ca^2+^ release events over the entire time course within the two ROIs are shown on the right. **B.** Same experimental conditions as in A, except that uncaging with the 405 nm laser was performed over the entire field continuously. The white box shows the localization of the first release event to an ER patch. **C–D.** **A single sub-threshold uncaging pulse results in preferential Ca^2+^ release from ER patches as compared to the reticular ER.** In this case a single uncaging pulse was applied (405 nm laser 50% power) only within the circular ROIs (white circles) as indicated by ‘Uncage’ on the traces in the lower panel. One ROI is over an ER patch and the other over the reticular ER. **D.** Same imaging conditions over a small area (inset, 9.15×3 µm) where a single uncaging pulse was applied over the entire imaged area. Ca^2+^ release was then analyzed from two region as indicated by the boxes representing an ER patch and the neighboring reticular ER. The lower traces show an expanded time scale of Ca^2+^ release.

In the first set of experiments we defined two regions of interest, one over an ER patch (P) and the other over the reticular ER (Ret), and imaged Ca^2+^ dynamics while continuously uncaging cIP_3_ in these ROIs using the 405 nm laser at low intensity to gradually buildup IP_3_ concentrations (Fig. 3A). An initial Ca^2+^ release spike is observed from the ER patch (Fig. 3A, marked b on the images & trace), followed by a larger Ca^2+^ release transient from the same patch (Fig. 3A, marked c at 28.86 sec). This larger Ca^2+^ release transient from the ER patch ROI results in a Ca^2+^ rise over a broad area that eventually diffuses to the reticular ER ROI (Ret). This results in a local Ca^2+^ rise over the reticular ER ROI (Fig. 3A, observed as a shoulder in the trace marked as d), which triggers Ca^2+^ release (Fig. 3A, panel e & trace).

Under this experimental paradigm IP_3_ is uncaged at the same rate in both the patch and reticular ER ROIs, arguing that IP_3_ receptors in both ROIs are subject to similar IP_3_ concentrations over a prolonged period of time (10–20 sec). Nonetheless, only IP_3_ receptors in the patch ER ROI respond by releasing Ca^2+^. IP_3_ receptors in the reticular ER ROI respond only when a local Ca^2+^ rise occurs due to the diffusion of Ca^2+^ released from the ER patch ROI. This fits with Ca^2+^ acting as a co-agonist of the IP_3_ receptor, where at a constant IP_3_ concentration a Ca^2+^ rise below a certain threshold increases the probability of opening of IP_3_ receptors, since IP_3_ receptor gating exhibits a bell-shaped dependence on Ca^2+^ [33], [34]. These results suggest that IP_3_ receptors in ER patches are more sensitive when compared to those in the reticular ER. Note that the amount of Ca^2+^ released from the reticular ER ROI is comparable to that observed over the ER patch ROI (Figure 3A, trace), arguing that local luminal Ca^2+^ content is not limiting.

We next replicated the conditions in the line scan protocol (Fig. 2B) by imaging Ca^2+^ transients while continuously uncaging IP_3_ at low amplitude across the entire field (Fig. 3B). This results in a propagative wave that is invariably initiated over an ER patch (Fig. 3B, white box) in all 12 cells tested. Therefore, as IP_3_ levels build up, Ca^2+^ release is preferentially triggered from ER patches as compared to the reticular ER, favoring the argument that IP_3_ receptors in ER patches are sensitized to IP_3_ compared to receptors in the reticular ER. In these experiments IP_3_ receptors in the two ER domains are exposed to similar IP_3_ concentration for several seconds. This prolonged exposure to IP_3_ shows that IP_3_ receptors in ER patches respond to sub-threshold IP_3_ concentrations that are incapable of inducing Ca^2+^ release from the reticular ER.

In the previous experimental approaches IP_3_ was gradually increased over time through continuous uncaging of cIP_3_. Given that IP_3_ can result in Ca^2+^-independent IP_3_ receptor inactivation [35], [36], the gradual slow increase in IP_3_ could potentially result in receptor inactivation, which may dampen Ca^2+^ release from the reticular ER. With this concern in mind, we designed experiments to define the sensitivity of IP_3_-dependent Ca^2+^ release in different ER domains following a single uncaging pulse within two ROIs (circles), one over an ER patch and the other over a reticular ER region (Fig. 3C). The uncaging pulse induced Ca^2+^ release from the ER patch but not the reticular ER ROI (Fig. 3C). This experiment was repeated on 11 cells with 8/11 showing Ca^2+^ release only from the ER patch domain (Fig. 3C), and 3/11 releasing Ca^2+^ from both the ER patch and reticular domain, albeit with a lower amplitude from the reticular domain. In a complementary approach a small region was scanned continuously and the entire imaged field stimulated by a single uncaging pulse (Fig. 3D). The expanded time scale and corresponding images show that Ca^2+^ release is initiated from the ER patch domain and spreads to the rest of the imaged field (Fig. 3D). This was observed in 14/17 cells, while 3/17 showed simultaneous release from both ER domains. Collectively these different approaches show that IP_3_ receptors within ER patches can sense and respond to IP_3_ concentrations not detectable by IP_3_ receptors in the neighboring reticular ER. This suggests that increasing IP_3_ receptor density due to ER remodeling sensitizes IP_3_-dependent Ca^2+^ release.

### Role of Ca^2+^-dependent cooperativity within ER patches in sensitizing IP_3_-dependent Ca^2+^ release

**Figure 4:**
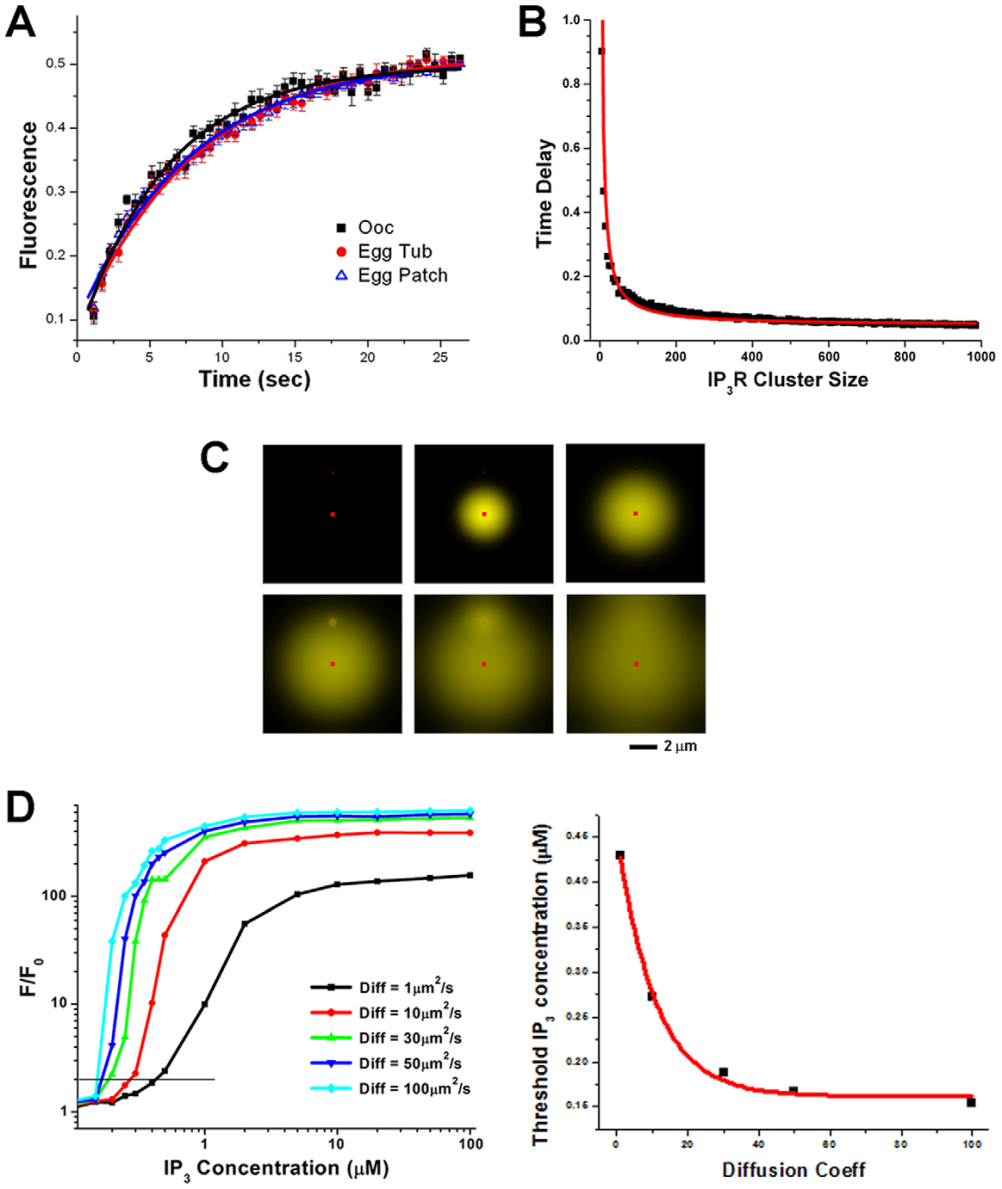
A. IP_3_ receptors lateral mobility in ER patches and reticular ER. Fluorescence recovery after photobleaching (FRAP) in oocytes and eggs expressing GFP-IP_3_ receptor (n = 7–8; mean±SE). The bleaching ROIs in eggs were positioned over ER patches or reticular ER. Recovery kinetics were fitted with a monoexponential decay function. Recovery kinetics show that the lateral mobility of IP_3_ receptors is comparable within ER patches and the reticular ER. **B–D.** **Mathematical modeling.** Modeling the time delay in Ca^2+^ release as a function of IP_3_ receptor cluster size. As IP_3_ receptor cluster size increases the delay in response to an IP_3_ pulse decreases. **C.** Modeling Ca^2+^ release spatial and amplitude distribution from two IP_3_ receptor clusters of varying size (20 and 1000) in response to a sub-threshold IP_3_ pulse. **D.** Simulation of the dependence of Ca^2+^ release within an IP_3_ receptor cluster as a function of IP_3_ concentration for varying Ca^2+^ diffusion coefficients. Lowering the Ca^2+^ diffusion coefficient results in less Ca^2+^-dependent cooperativity between neighboring receptors. Decreasing Ca^2+^ diffusion coefficient leads to decreased sensitivity of IP_3_ receptors as illustrated by the threshold IP_3_ concentration required to initiate Ca^2+^ release.

How can the sensitivity of IP_3_ receptors in distinct ER sub-domains be modulated differentially? One possibility is that there exists a different population of IP_3_ receptors in ER patches versus the reticular ER with differential posttranslational modifications and/or protein-protein interactions. To test whether this is the case we measured the mobility of GFP-tagged IP_3_ receptors in the two ER sub-domains using fluorescence recovery after photo-bleaching (FRAP) (Fig. 4A). The rate of recovery from photo-bleaching was indistinguishable in ER patches and the reticular ER, and was comparable to that of IP_3_ receptors in oocytes before ER remodeling (Fig. 4A). This argues that IP_3_ receptors' lateral mobility is equivalent between the different ER subdomains. However, the recovery from photobleaching was not complete (Fig. 4A) showing that a sub-population of IP_3_ receptors in immobile. The immobile fraction of IP_3_ receptors in oocytes (57.14±0.86), egg reticular ER (56.5+0.86) and egg ER patches (57.61+0.57) is also similar (n = 14–21). Given the percent immobile fraction it is possible that a sub-population of IP_3_ receptors localize differentially to ER compartments. This is unlikely though, because ER patches are continuously remodeling and fusing with reticular ER domains (Fig. 2A), arguing that the same population of IP_3_ receptors exists in ER patches and reticular ER domains.

We then considered the possibility that the density of IP_3_ receptors in the different ER sub-domains modulates their sensitivity as a population. The probability of opening (P_o_) of IP_3_ receptors exhibits a bell-shaped dependence on Ca^2+^ [33], [34], which plays a role in the cooperativity of gating of IP_3_ receptors within a cluster [4], [37], [38]. At the ultrastructural level ER patches in the egg are formed by a complex three dimensional ER rearrangement (Fig. S2) [17], which brings IP_3_ receptors in close physical proximity. Therefore, increased density of IP_3_ receptors within an ER patch could cooperatively increase their P_o_, leading to functional sensitization. To determine whether this is the case we modeled the time delay in Ca^2+^ release as a function of IP_3_ receptor cluster size, which shows an inverse relationship (Fig. 4B). Therefore, the higher the density of IP_3_ receptors (large cluster) the shorter the time delay in responding to IP_3_ to gate the receptor open (Fig. 4B). This is consistent with IP_3_ receptor sensitization due to increased density. We then modeled the response of two IP_3_ receptor clusters of different sizes (20 or 1000 receptors) (Fig. 4C). Simulating an IP_3_ rise across the entire field leads to Ca^2+^ release from the large IP_3_ receptor cluster, which diffuses to the smaller cluster leading to Ca^2+^ release (Fig. 4C), replicating the experimental observation in Figure 3A, and showing that IP_3_ receptors within the large cluster are more sensitive to IP_3_. Details of the mathematical model are discussed in Appendix S1.

We then tested whether Ca^2+^-dependent cooperativity within a large cluster of IP_3_ receptors modulates their sensitization. To alter Ca^2+^-dependent cooperativity we varied Ca^2+^ diffusion coefficient, reasoning that higher Ca^2+^ diffusion coefficients increase IP_3_ receptors cooperativity by enhancing cross-talk (Ca^2+^-dependent gating) between channels within a cluster. Changing the Ca^2+^ diffusion coefficient will not affect IP_3_ receptor affinity to its ligand IP_3_. A dose response relationship of IP_3_-dependent Ca^2+^ release as a function of IP_3_ concentrations shows a shift to the right as Ca^2+^ diffusion coefficient decreases (Fig. 4D). From this plot one can estimate the sensitivity of IP_3_-dependent Ca^2+^ release as the threshold IP_3_ concentration required to produce Ca^2+^ release (F/F_0_), in this case set as double the resting Ca^2+^ (Fig. 4D, left panel). The threshold IP_3_ concentration decays exponentially as a function of the Ca^2+^ diffusion coefficient (Fig. 4D, right panel). This supports a model, where clustering of IP_3_ receptors within the three dimensional space of an ER patch enhances channel cooperativity leading to their sensitization. The sensitization of IP_3_ receptors in this case is a population property that is independent from the IP_3_ affinity of each individual IP_3_ receptor. These results show that ER remodeling tunes the sensitivity of IP_3_ receptors by increasing their density and as such Ca^2+^-dependent cooperativity. When IP_3_ receptors are concentrated in a three-dimensional volume as in the context of an ER patch, stochastic opening of individual receptors would lead to Ca^2+^-dependent sensitization of neighboring receptors resulting in Ca^2+^ release.

### ER remodeling modulate Ca^2+^ wave propagation

**Figure 5:**
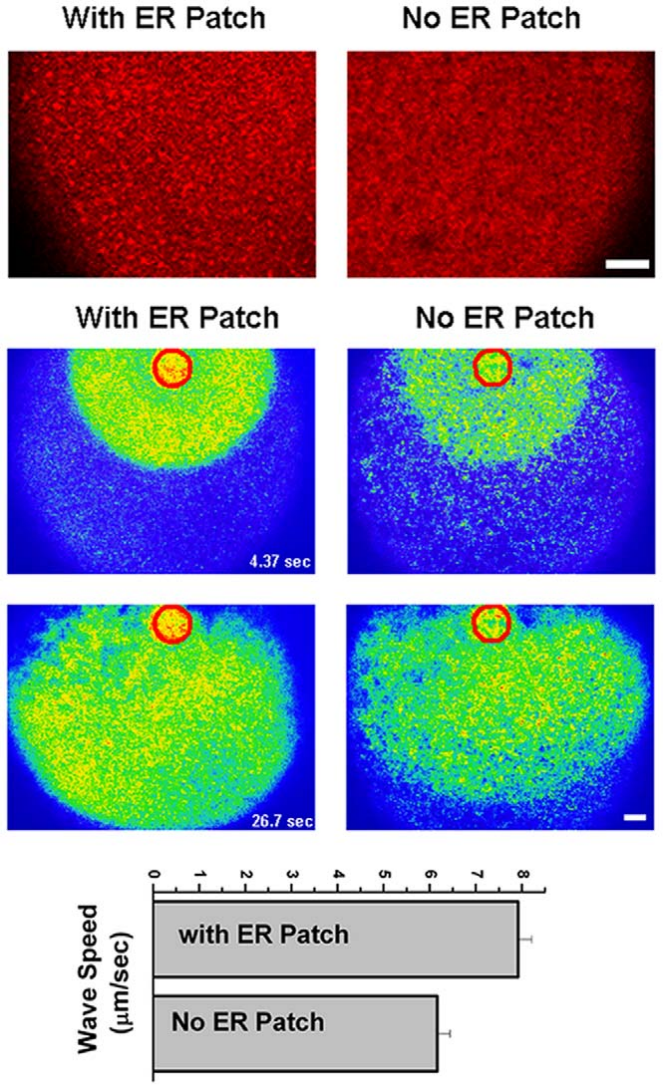
Role of ER remodeling in Ca^2+^ wave propagation. Cells expressing mCherry-KDEL to visualize ER distribution were injected with caged-IP_3_ and Oregon-Green to image Ca^2+^ dynamics. Top panel shows an enlarged region from two cells, one with ER patches and one without. Ca^2+^ waves were induced by uncaging IP_3_ within an ROI (red circle) and wave propagation speed was measured. Example images of wave propagation are shown. These experiments were performed on the animal hemisphere (n = 12–13).

To test the role of ER patches in Ca^2+^ wave propagation we measured the speed of propagation of the Ca^2+^ wave in eggs with or without ER patches on the animal hemisphere. As shown in Figure 1C a small percentage of eggs exhibit no ER patches. We tried to increase this percent by treating cells with nocodazole, however found that when cells were injected with Oregon-green, nocodazole was no longer effective at preventing patch formation. The reasons for this observation are unclear but could be due to a Ca^2+^-dependence of nocodazole action on microtubules. Nonetheless, the natural diversity of patch occurrence in individual oocytes allowed us to test whether the presence of ER patches has any effect on the speed of propagation of the Ca^2+^ wave. An uncaging pulse on the animal hemisphere leads to a global Ca^2+^ wave in both cells with or without ER patches (Fig. 5). However, the speed of propagation was significantly slower is cells with no ER patches (p = 1.2×10^−4^) (Fig. 5), arguing that ER patches are important in modulating Ca^2+^ wave speed.

### Role of the oocyte maturation kinase cascade in ER remodeling and IP_3_ receptor sensitization

**Figure 6:**
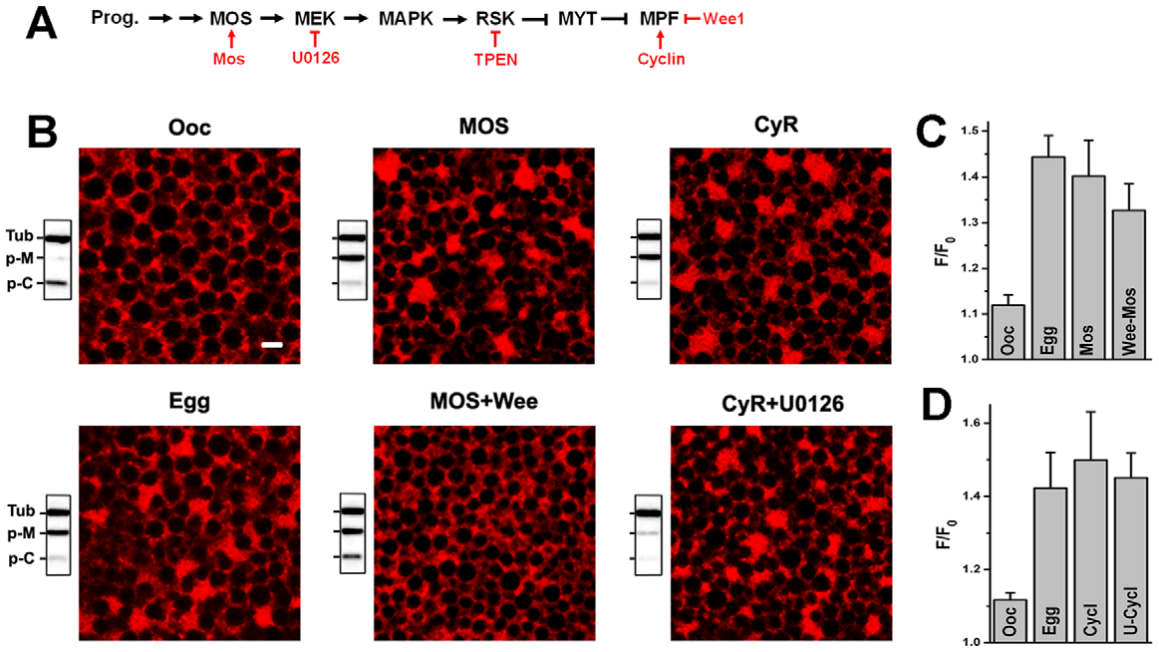
MPF is required for ER remodeling during meiosis. **A.** Simplified kinase cascade activated during oocyte maturation. Indicated in red is the action of different molecular and pharmacological modulators. The arrow indicates activation and the bar inhibition. **B.** Confocal fields from individual cells expressing mCherry-KDEL and treated as indicated showing ER structure. After imaging cells were lysed and subjected to Western blotting analysis for phosphor-MAPK (p-M), phosphor-Cdc2 (p-C) and tubullin (Tub) as the loading control (Scale bar, 2 µm). Mos indicates cells injected with Mos RNA, CyR: Cyclin B1 RNA; MOS+Wee: inject Wee1 RNA and incubate cells overnight before Mos RNA injection; CyR+U0126: pre-treat with U0126 (50 µM) for 1 h before Cyclin B1 RNA injection. The effectiveness of these treatments is illustrated in Western blots performed on individual oocytes after confocal imaging. The top band (Tub) represents the tubulin loading control, the middle band phosphorylated MAPK (p-M) and the lower band phosphorylated cdc2 at Tyr-15 (p-C). MAPK phosphorylation indicates its activation, whereas the phosphorylation of Cdc2, the kinase subunit of MPF, indicates its inhibition. **C–D.** Sensitivity of IP_3_-dependent Ca^2+^ release measured as indicated in the text.

In both *Xenopus* and mouse oocytes the kinase cascade driving maturation plays an important role in the sensitization of IP_3_-dependent Ca^2+^ release [24]–[26]. In *Xenopus*, sensitization of Ca^2+^ release correlates with the activation of MPF and hyper-activation of either the MAPK cascade or MPF is sufficient to sensitize Ca^2+^ release [24]. The IP_3_ receptor is phosphorylated at consensus MPF/MAPK sites during oocyte maturation [24]. Furthermore, modeling studies suggest that increased IP_3_ receptor affinity is a determinant of the changes observed in both elementary and global Ca^2+^ signaling during oocyte maturation [27]. This raises questions regarding the relative contribution of the oocyte maturation kinase cascade, ER remodeling and IP_3_ receptor phosphorylation to IP_3_ receptor sensitization. To address this issue, we correlated Ca^2+^ release sensitivity with the activation state of the kinase cascades and the formation of ER patches. To assess the sensitivity of IP_3_-dependent Ca^2+^ release a threshold uncaging pulse was empirically defined in oocytes and applied to eggs (Fig. 6). Normalization of the data to the Ca^2+^ release levels in oocytes provides a measure of the sensitization of Ca^2+^ release in response to IP_3_ [24].

Modulation of the kinase cascade by different molecular or pharmacological regulators allows differential activation of kinases along the cascade as summarized in Figure 6A. In contrast to oocytes, in eggs MAPK and MPF are active, ER patches are apparent and IP_3_-dependent Ca^2+^ release is sensitized (Fig. 6B–D, Egg). Expressing Mos or Cyclin B (CyR) activates both MAPK and MPF, and results in ER patch formation and sensitization of Ca^2+^ release (Fig. 6B–D). Activation of MPF independently of MAPK using the Cyclin B-U0126 approach (Fig. 6A), results in ER patch formation and sensitization of Ca^2+^ release (Fig. 6B & D), showing that MPF activation is sufficient to remodel the ER during meiosis. This is confirmed by activation of the MAPK cascade independently of MPF, using Wee1 followed by Mos expression, which does not result in ER reorganization into patches (Fig. 6B). However, in this case Ca^2+^ release is still sensitized (Fig. 6C), as previously shown [24]. These data argue that ER remodeling during meiosis requires MPF, the master kinase that drives the cell division phase. However, the sensitization of IP_3_-dependent Ca^2+^ release still occurs in the absence of ER remodeling when the MAPK cascade is hyper-activated, suggesting that both ER remodeling and phosphorylation of either the IP_3_ receptor, or other effectors that modulate IP_3_ receptor activity, contribute to sensitization of IP_3_-dependent Ca^2+^ release during meiosis. As such they may represent redundant mechanisms that ensure IP_3_ receptor sensitization, and egg activation at fertilization.

## Discussion

The IP_3_ receptor transduces signals downstream of PLC-coupled receptors into Ca^2+^ transients, and acts as a central signal integrator through its large cytoplasmic domain, which interacts with multiple modulators [39]. Ca^2+^ released through IP_3_ receptors plays critical cellular functions, including the regulation of gene expression, cell growth and secretion. IP_3_ receptors coalesce into clusters of several IP_3_ receptors, which underlie elementary Ca^2+^ release events. The size of these elementary events depends on the number of active receptors, which is regulated by cross talk between IP_3_ receptors within a cluster through Ca^2+^-dependent potentiation or potentially direct protein-protein interaction [4]–[6]. Whether the size and function of these clusters is modulated by the ligand IP_3_ is a matter of debate [40], [41]. In addition to this steady-state physiological clustering of IP_3_ receptors, significantly larger clusters detectable by fluorescence microscopy (0.6–1 µm), are induced following sustained stimulation of Ca^2+^ signaling [10]–[12], however the physiological significance of this clustering is unclear. These large IP_3_ receptor aggregations appear to be due to lateral diffusion of IP_3_ receptors and protein-protein interactions, and do not involve changes in ER structure [10]–[12]. In contrast, the clustering of IP_3_ receptors observed during oocyte meiosis is due to restructuring of the ER. We show that ER remodeling during *Xenopus* oocyte meiosis requires MPF activation, consistent with results in mouse [31] and nemertean eggs [42]. Furthermore, MPF activation has been shown in several species to be important for Ca^2+^ signaling differentiation during oocyte maturation [2], [22], [24], [43]–[45].

In the context of oocyte maturation in preparation for fertilization, IP_3_-dependent Ca^2+^ release is sensitized and contributes to the generation of the specialized Ca^2+^ transient, which is essential for egg activation [2]. Remodeling of the ER during oocyte meiosis is well documented in different species, and ER patches are enriched in the egg's cortex [17], [32], [46]–[49]. The formation of such ER patches correlates with the sensitization of IP_3_-dependent Ca^2+^ release [17], [28], [32], and as such they have been postulated as initiation sites for repetitive Ca^2+^ waves in mouse, and as important for wave propagation in *Xenopus* [17], [30]. Furthermore, the tight correlation between ER reorganization and Ca^2+^ signaling remodeling during oocyte maturation suggests a has (reviewed in [30]. However, the mechanisms underlying a potential role for ER restructuring during meiosis in modulating Ca^2+^ signaling are unknown. Here we show that the remodeling of the ER underlies the clustering of elementary Ca^2+^ release events observed during meiosis [15], since the large elementary Ca^2+^ release events in the egg (SREs) localize to ER patches (Fig. 2). Furthermore, ER remodeling also affects global Ca^2+^ dynamics in the egg by modulating the propagation speed of the Ca^2+^ wave in the egg (Fig. 5), which mediates egg activation events such as the block to polyspermy and completion of meiosis [2]. Hence ER remodeling modulates both elementary and global Ca^2+^ dynamics and as such plays a central role in endowing the egg with the competency to activate at fertilization.

An important aspect of Ca^2+^ signaling remodeling during oocyte maturation is the sensitization of IP_3_-dependent Ca^2+^ release, the main pathway underlying the Ca^2+^ transient at fertilization [2]. We show that ER remodeling contributes to sensitization of IP_3_-dependent Ca^2+^ release through a simple and elegant mechanism that we refer to as ‘geometric sensitization’. In the context of ER patches formed during meiosis, IP_3_ receptors are sensitized as a population (Fig. 3) apparently due to their increased density in the three-dimensional space of the ER patch (Fig. 4). The packing of membranes in ER patches brings IP_3_ receptors into close proximity (40–50 nm) (Fig. S2). This is predicted to enhance Ca^2+^-dependent cooperativity between neighboring receptors. Given the bell-shaped dependence of IP_3_ receptor gating on Ca^2+^ and the fact that Ca^2+^ acts as a co-agonist for the IP_3_ receptor, increased Ca^2+^-dependent cooperativity between IP_3_ receptors within an ER patch could sensitize them to respond to sub-threshold IP_3_ concentration as observed in mature eggs. Mathematical modeling supports this conclusion. Simply increasing Ca^2+^-dependent cooperativity between IP_3_ receptors in a cluster sensitize them to gate open at lower IP_3_ concentration (Fig. 4). We altered Ca^2+^-dependent cooperativity between IP_3_ receptors by modulating the Ca^2+^ diffusion coefficient in a cluster of IP_3_ receptors to represent the context of an ER patch (Fig. 4). This is consistent with what is observed experimentally, where IP_3_ receptors within an ER patch can sense and gate open in response to IP_3_ concentrations that are undetectable by IP_3_ receptors in the reticular ER (Fig. 3).

Therefore, ‘geometric sensitization’ of IP_3_ receptors due to ER remodeling represents a physiological modulator of IP_3_ receptor function during meiosis. This mechanism could be applicable to other physiological situations as during mitosis where the ER restructures with the preponderance of the evidence arguing for a transition from a tubular to a cisternae-like structures in mitosis [50]–[52].

In summary we show that the physical structure of the ER has a profound effect on IP_3_ receptor function. During oocyte meiosis ER remodeling regulates both elementary and global aspects of Ca^2+^ signaling and is involved in sensitizing IP_3_ receptors. We argue that ER patch formation during meiosis increases the density of IP_3_ receptors thus enhancing Ca^2+^-dependent cooperativity. This leads to sensitization of IP_3_ receptors allowing them to gate open at sub-threshold IP_3_ concentrations. IP_3_ receptor sensitization is central to producing the fertilization specific Ca^2+^ transient, and as such for egg activation. Therefore, ER remodeling through geometric sensitization of IP_3_ receptors plays a central role in oocyte maturation.

## Materials and Methods

### Ethics Statement

All studies were conducted in accordance with the National Institutes of Health Guide for the Care and Use of Laboratory Animals and in accordance with Qatari regulations regarding the use of animal subjects in research as outlined by the Supreme Council of Health. Studies were approved by the Institutional Animal Care and Use Committee (IACUC) of Weill Cornell Medical College protocol #0806-759A.

### Molecular Biology

pSp64s-GFP-KDEL was a gift from Mark Terasaki [17]. pGEM-HE-NOT-GFP-rIP3R1 was generous gift from Patricia Camacho. pSGEM-mCherry-KDEL and pSGEM-mCherry-STIM1 plasmids were previously described [23], [53]. All constructs were verified by DNA sequencing and by analytical endonuclease restriction enzyme digestion. For *in vitro* transcription, after linearization with NheI or NotI, capped RNAs were transcribed using T7 RNA polymerase with T7 mMESSAGE mMACHINE kit (Ambion). *Xenopus* oocytes were obtained as described previously [22]. Oocytes injected with the different RNAs were incubated either at room temperature or 18°C for 1–3 days. Oocytes injected with GFP-rIP3R1 RNA required significantly longer time to express the GFP-tagged IP_3_ receptor and were kept at 18°C for 7–9 days depending on the batch of oocytes before analysis. For experiments aimed at modulating the kinase cascade differentially in the oocyte (Figure 6), cells were incubated for a few hours after Mos or cyclin B RNA injection until they resulted in GVBD as indicated by the appearance of a white spot on the animal pole. Wee1 RNA was injected 12–16 hours before Mos injection [22]. The plasmid used as templates to produce Mos, Wee and Cyclin B RNA were as previously described [22]. For the U0126-cyclib B treatment cells were preincubated with U0126 (50 µM) for 1 hour before injecting Cyclin B RNA.

### Imaging

Live cell imaging was performed on a Zeiss LSM710 confocal using a Plan Apo 63×/1.4 oil DIC II objective. The image size was typically set at 744×744 pixel (67.38×67.38 µm) with pinhole set at 1 airy unit (0.9 µm) unless otherwise specified. GFP and Oregon-Green signals were excited with 488 nm laser and emissions collected through a bandwidth of 492–558 nm, mCherry was excited at 561 nm and emission collected through a bandwidth of 572–699 nm. Cells were imaged in OR2 solution (in mM: 82.5 NaCl, 2.5 KCl, 1 CaCl2, 1 Na2HPO4, 5 HEPES, pH 7.5). Images were analyzed using ZEN 2008 and MetaMorph and figures compiled using Adobe Photoshop.

For FRAP analyses pre-bleach and recovery images were scanned (pixel dwell time: 0.84 µs) at 561 nm with 1% laser power. A 25×25 pixels area was bleached at 60% laser power at reduced scanning speed (pixel dwell time: 5.31 µs). FRAP recovery curves were derived by comparison with reference unbleached area, subtracting the background, and fitting with a monoexponential decay function.

Ca^2+^ waves were analyzed in eggs expressing mCherry-KDEL and injected with caged-IP_3_ (NPE-caged inositol 1,4,5-trisphosphate (Invitrogen I23580) and 40 µM Oregon Green 488 BAPTA-1 hexapotassium salt (Invitrogen O6806). Cells were scanned in Ca^2+^-free OR2 solution using Plan-Neofluar 40×/1.3 oil DIC objective with 488 and 561 nm laser set at 2% intensity. The image size is 256×256 pixel (352×352 µm) with pinhole set at 4 µm. After 5 initial scans, an circular ROI (diameter 30 µm) was bleached 50 times with the 405 nm laser set at 20% power resulting in propagating Ca^2+^ wave that crossed the entire cell.

### Immunoblot Analysis

Phospho-MAPK and phospho-Cdc2 Westerns were performed as described previously [54], and α-tubulin was used as the loading control.

### Mathematical Modeling

The details of the model used are included in Appendix S1.

## Supporting Information

Figure S1 **ER patch spatial distribution.** **A.** ER patches form in GFP-KDEL expressing eggs. Planes at which confocal images were taken along z-stack are indicated by the matching number in the orthogonal z-section. **B.** Images from eggs expressing mCherry-KDEL at GVBD which were either untreated (Egg Con) or treated with Nocodazole (25 µg/ml) for 1 hr.
(TIF)

Figure S2 **Low (A) and high (B) magnification transmission EM images of ER patches in a** ***Xenopus*** **egg.** Patches in the low magnification image are highlight by the white contour.
(TIF)

Appendix S1 **Computational Modeling.**
(DOCX)
