# Supplementary figures and images for: Endoplasmic Reticulum Remodeling Tunes IP3-Dependent Ca2+ Release Sensitivity
Source: PLoS One. 2011 Nov 30;6(11):e27928. doi: 10.1371/journal.pone.0027928 (PMC3227640; doi:10.1371/journal.pone.0027928)

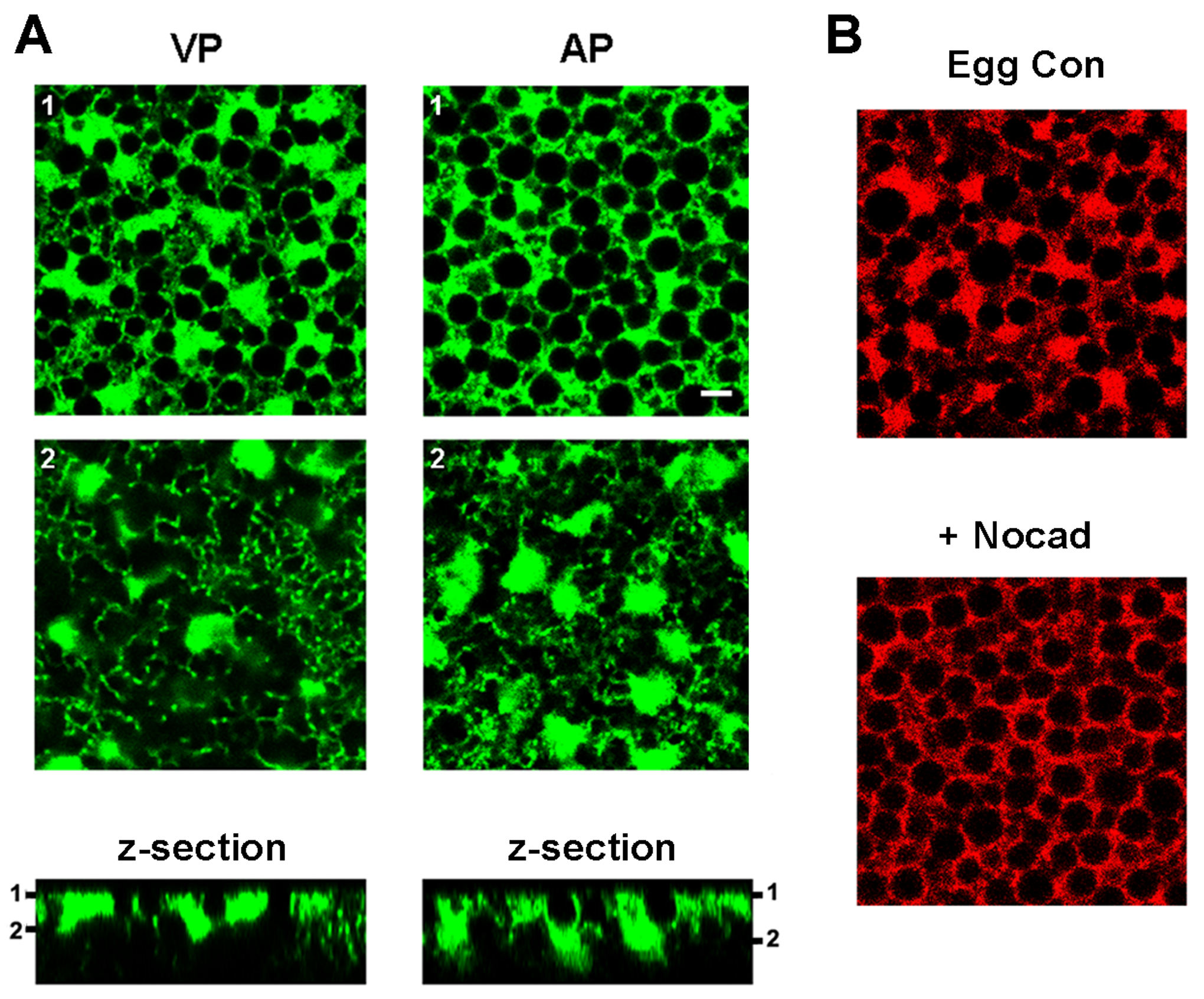

Supplement: Figure S1 — ER patch spatial distribution. A. ER patches form in GFP-KDEL expressing eggs. Planes at which confocal images were taken along z-stack are indicated by the matching number in the orthogonal z-section. B. Images from eggs expressing mCherry-KDEL at GVBD which were either untreated (Egg Con) or treated with Nocodazole (25 µg/ml) for 1 hr. (TIF) [file pone.0027928.s001.tif]

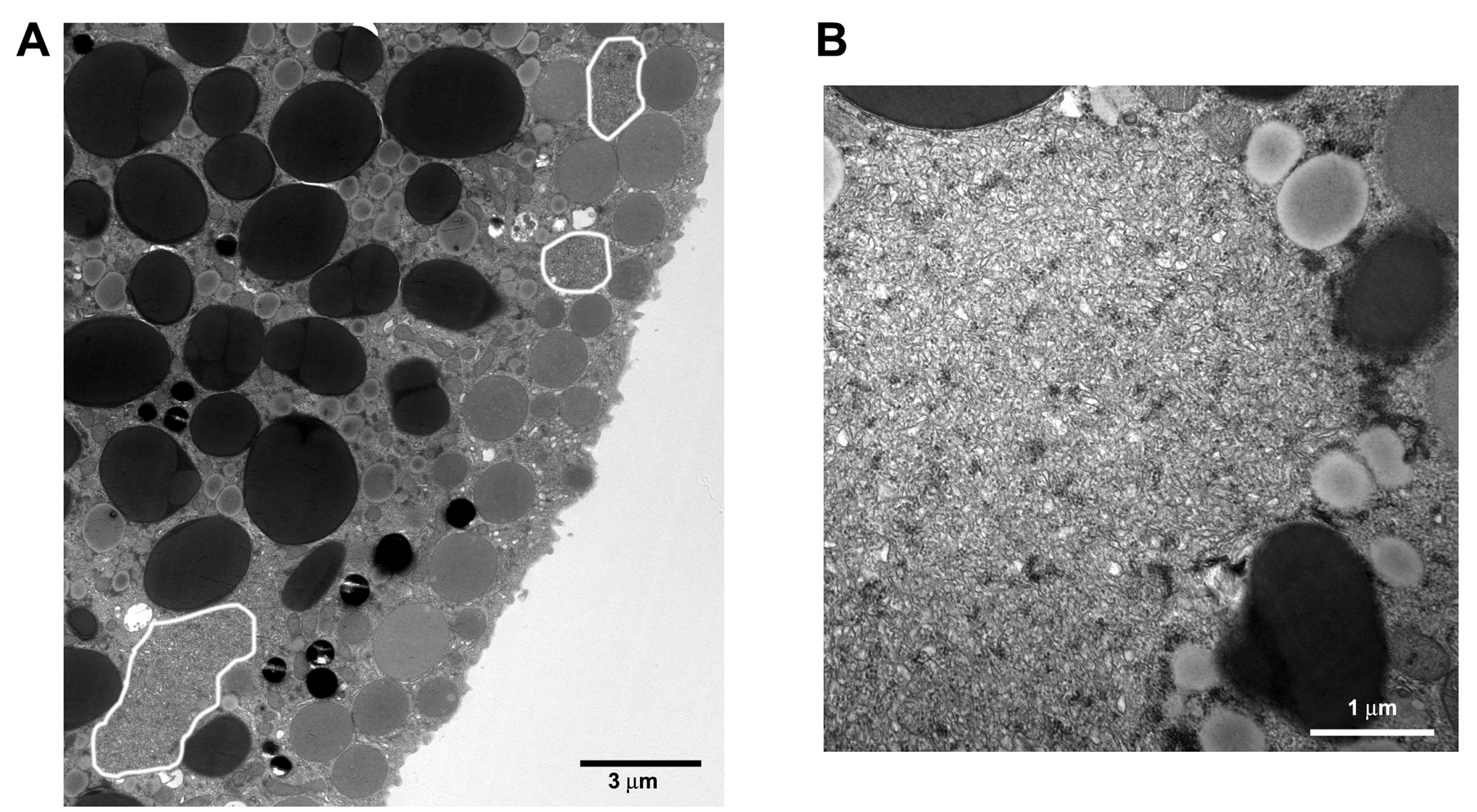

Supplement: Figure S2 — Low (A) and high (B) magnification transmission EM images of ER patches in a Xenopus egg. Patches in the low magnification image are highlight by the white contour. (TIF) [file pone.0027928.s002.tif]
