# Supplementary material for: Endoplasmic Reticulum Remodeling Tunes IP3-Dependent Ca2+ Release Sensitivity
Source: PLoS One. 2011 Nov 30;6(11):e27928. doi: 10.1371/journal.pone.0027928 (PMC3227640; doi:10.1371/journal.pone.0027928)
Supplement: Appendix S1 — Computational Modeling. (DOCX) [file pone.0027928.s003.docx]

**Supplemental Material**

**Appendix: Computational Modeling**

We model the release of calcium from ER through IP3 channels in various spatial configurations. The focus of this exploration is on the effects of spatial organization and the chosen detail of the model reflects this focus.

We consider a patch of membrane which separates the ER from the cytosol. The spreading of calcium in the cytosol is described by the following reaction diffusion equation (see e.g. Shuai and Jung [1])

$$\frac{dc}{dt}=D\nabla^{2}c+J_{IP3Rs}+J_{leak}\left( c \right)-J_{SERCA}(c)$$

(Eq.1)

where c denotes the cytosolic calcium concentration and D the effective diffusion coefficient, which incorporates the effect of calcium buffers [2]. Calcium is released into the cytosol through IP_3_Rs, distributed in arbitrary geometric arrangements as single channels or clusters. Such calcium sources are mathematically described by

$$J_{IP3Rs}= \sum_{i} \pi r^{2}N_{open}(i,t)v_{channel}(c_{ER}-c)\delta(\vec{x}-\vec{x}_{i})$$

(Eq.2)

where the delta-functions describes the location of the i’th channel/cluster, N_open_(i,t) the number of open channels in that cluster at time t, and r the radius of each channel. Release, diffusion and hydrolysis of IP_3_ (denoted by p) is described by

$$\frac{\partial p}{\partial t}= -\left( p-p_{0} \right)\gamma_{d}+j_{in}(t)$$

(Eq.3)

where p_0_ denotes the baseline concentration, γ_d_ its decay rate, and the time course of global stimulation through IP_3_-uncaging. Calcium is pumped back into the ER by SERCA described by the Hill form

$$J_{SERCA}= v_{SERCA}\frac{c^{2}}{k_{p}^{2}+c^{2}}$$

(Eq.4)

and escapes the ER through nonspecific leaks described by

$$J_{leak}=v_{leak}(c_{ER}-c)$$

(Eq.5)

To simulate the gating of each IP_3_ receptor in order to obtain the number of open channels, we use a simple stochastic model which has been put forward and tested against experimental data in (Swimanathan et al. [3]). The parameters in Eqs 1-5 are given in Table 1.

| Parameter | Value |  |
| --- | --- | --- |
| c_ER_ | 500μM | ER calcium concentration |
| v_channel_ | 318/s | channel flux |
| v_leak_ | 0.002/s | leakage conductance |
| v_pump_ | 40μM/s | Max SERCA flux |
| r | 10nm | channel radius |
| k_p_ | 0.18μM | SERCA disassociation constant |
| D | 25μm^2^/s | effective Ca-diffusion coefficient |

The set of differential equations Eqs. 1,3 are solved in two dimensions using a standard first order, fully explicit method with a 4-point discretization of the Laplacian. The time step dt and the spatial discretization h have been chosen appropriately to guarantee convergence and to sample the kinetics of the IP_3_Rs sufficiently frequently. To implement the source terms, we use for each source a discrete implementation of the δ-function, i.e.

$$\delta\left( x-x_{0} \right)\delta\left( y-y_{0} \right)=\left\{ \begin{aligned} 0 for x\neq x_{0}, y\neq y_{0} \\ 1/h^{2} for x=x_{0}, y=y_{0} \end{aligned} \right.$$

(Eq.6)

**A.1 Response delay and cluster size**

We position a cluster of N channels in the center of a 5μm x 5μm membrane. Initially, the IP_3_ concentration is zero, all channels are closed and cytosolic calcium is at its baseline concentration determined by SERCA and the leak currents. Then we mimic rapid global stimulation by rapidly increasing overall IP_3_ levels to 2μM. After some delay, t_d_(N), the cluster will fire a first puff (defined by the first opening of at least 50% of the channels). These delay times are averaged over 100 identical runs with reseeded random number generators. The average delay time between the stimulation and the first puff is plotted as a function of the cluster size in Fig. 3. The delay time decreases with increasing cluster size. The curve can be fitted to

$$\left( t_{d}\left( N \right) \right)=(\frac{A}{N}+B)e^{-CN}$$

(Eq.7)

with A=4.1,B=1.08 and C=3.7E-05.

In Fig. 3 we show snapshots of a simulation, where we place a large cluster of 1000 channels in the center of a 10μm x 10μm membrane and a small cluster of 20 channels at 3μm distance. As one expects from Fig.1, the large cluster fires a puff first and the released calcium, as it reaches the small cluster, aids the release of a puff through calcium-induced calcium release.

**A.1 Gating cooperativity increases sensitivity**

To demonstrate that channel clustering increases effectively the sensitivity of the calcium signaling machinery via gating cooperativity, we simulate a large group of single IP_3_ channels at a distance of 0.5μm and vary the diffusion coefficient D (see Eq.1). For a small diffusion coefficient, calcium release from one channel will affect the neighbor channels very little and the observed overall calcium response is from independent channels. Increasing the diffusion coefficient, calcium release from one channel reaches nearby channels introducing gating cooperativity through calcium-induce calcium release.

We start initially at zero IP_3_ concentration and baseline calcium concentration and rapidly increase global ip3 concentration. As a measure of the sensitivity of the calcium signaling machinery, we use the maximum field-averaged calcium concentration within 10s after stimulation divided by the baseline calcium concentration. The resulting dose-response curves are shown in Fig. 3. For increasing diffusion coefficients D, i.e. gating cooperativity, the threshold of detectability moves dramatically to smaller values of IP_3_ although the total number of channels has not changed.

Reference List

1. Shuai JW, Jung P (2003) Optimal ion channel clustering for intracellular calcium signaling. Proc Natl Acad Sci U S A 100: 506-510.

2. Yao Y, Choi J, Parker I (1995) Quantal puffs of intracellular Ca2+ evoked by inositol trisphosphate in Xenopus oocytes. J Physiol 482 ( Pt 3): 533-553.

3. Swaminathan D, Ullah G, Jung P (2009) A simple sequential-binding model for calcium puffs. Chaos 19: 037109.
